# Skin-inspired hydrogel–elastomer hybrids with robust interfaces and functional microstructures

**DOI:** 10.1038/ncomms12028

**Published:** 2016-06-27

**Authors:** Hyunwoo Yuk, Teng Zhang, German Alberto Parada, Xinyue Liu, Xuanhe Zhao

**Affiliations:** 1 Department of Mechanical Engineering, Soft Active Materials Laboratory, Massachusetts Institute of Technology, Cambridge, Massachusetts 02139, USA; 2 Department of Mechanical and Aerospace Engineering, Syracuse University, Syracuse, New York 13244, USA; 3 Department of Chemical Engineering, Massachusetts Institute of Technology, Cambridge, Massachusetts 02139, USA; 4 Department of Civil and Environmental Engineering, Massachusetts Institute of Technology, Cambridge, Massachusetts 02139, USA

## Abstract

Inspired by mammalian skins, soft hybrids integrating the merits of elastomers and hydrogels have potential applications in diverse areas including stretchable and bio-integrated electronics, microfluidics, tissue engineering, soft robotics and biomedical devices. However, existing hydrogel–elastomer hybrids have limitations such as weak interfacial bonding, low robustness and difficulties in patterning microstructures. Here, we report a simple yet versatile method to assemble hydrogels and elastomers into hybrids with extremely robust interfaces (interfacial toughness over 1,000 Jm^−2^) and functional microstructures such as microfluidic channels and electrical circuits. The proposed method is generally applicable to various types of tough hydrogels and diverse commonly used elastomers including polydimethylsiloxane Sylgard 184, polyurethane, latex, VHB and Ecoflex. We further demonstrate applications enabled by the robust and microstructured hydrogel–elastomer hybrids including anti-dehydration hydrogel–elastomer hybrids, stretchable and reactive hydrogel–elastomer microfluidics, and stretchable hydrogel circuit boards patterned on elastomer.

## Abstract (web summary)

Soft hybrids that integrate hydrogels and elastomers can be used in applications, such as stretchable electronics and soft robotics, but usually have shortcomings. Here, Zhao and co-workers show a simple method of assembling hydrogel/elastomer hybrids with robust interfaces and functional microstructures.

## Graphical abstract

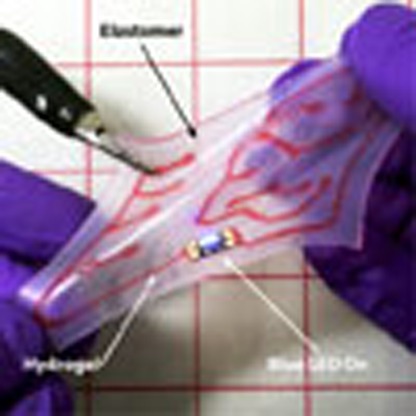

Soft materials including elastomers and hydrogels have enabled diverse modern technologies including tissue engineering^1^,^2^, drug delivery^3^, biomedical devices^4^,^5^, microfluidics^6^,^7^, optics^8^,^9^,^10^, stretchable and bio-integrated electronics^11^,^12^,^13^,^14^, and soft robotics^15^,^16^. Whereas elastomers have unique characters such as stable in various environments, mechanically robust and easy for micro-/nano-scale fabrications (for example, soft lithography); hydrogels' distinctive attributes include high water contents, permeable to various chemical and biological molecules, biocompatible and/or biodegradable. Since the merits of elastomers and hydrogels are complementary to each other, it is naturally desirable to integrate them into hybrid structures that can potentially transform their existing applications and enable new functions^17^,^18^,^19^,^20^,^21^. In nature, mammalian skins laminate elastomer-like epidermis and hydrogel-like dermis into hybrids with robust interfaces (for example, interfacial toughness over 100 Jm^−2^) (ref. 22) and functional microstructures (for example, blood and lymphatic vessels). However, elastomers and hydrogels in most technological applications are used separately^2^,^3^,^4^,^5^,^6^,^7^,^8^,^9^,^10^,^11^,^12^,^13^,^14^,^15^,^16^, and few existing hydrogel–elastomer hybrids suffer from limitations such as weak interfacial bonding, low robustness and difficulties in patterning microstructures^17^,^18^,^19^.

Tough bonding of hydrogels to rigid solids (for example, glass, ceramics and metals) have been recently achieved by covalently crosslinking the stretchy polymer networks of tough hydrogels on surfaces of the solids^23^. However, this method is generally inapplicable in forming hydrogel–elastomer hybrids with robust interfaces and functional microstructures, mainly due to three challenges: First, elastomers are highly permeable to oxygen that leads to the oxygen inhibition effect by which the free-radical polymerization or surface covalent crosslinking of hydrogel polymers are seriously hampered^24^. Second, aging or hydrophobic recovery of functionalized elastomer surfaces also significantly lower the effectiveness of hydrogel bonding on elastomers^25^. Third, most elastomers and hydrogels are fabricated by curing pre-elastomer resins and pre-gel solutions, and the pre-gel solutions (or pre-elastomer resins) can infiltrate into microstructures patterned on cured elastomers (or hydrogels) to diminish microstructures such as micro-channels^26^. A general method capable of fabricating hydrogel–elastomer hybrids with robust interfaces and functional microstructures is still a critical demand and central challenge in the field.

Inspired by the structures and functions of mammalian skins, here we report a simple yet general method capable of assembling pre-shaped elastomers and hydrogels into hybrid structures with extremely robust interfaces (for example, interfacial toughness over 1,000 Jm^−2^) and functional microstructures (for example, micro-channels and circuit patterns). The new method addresses the abovementioned challenges by integrating three innovations in fabrication of soft materials and soft hybrids. First, physical crosslinking of dissipative polymer networks in tough hydrogels to set their shapes and microstructures. Second, modification of cured elastomer surfaces with benzophenone to alleviate oxygen inhibition effect and activate elastomer surfaces for hydrogel polymer grafting. Third, covalent crosslinking of stretchy polymer networks in pre-shaped hydrogels on elastomers to give extremely robust and microstructured interfaces. The method is generally applicable to various types of commonly used elastomers including polydimethylsiloxane Sylgard 184, polyurethane, latex, VHB and Ecoflex, and diverse tough hydrogels including PAAm-algiante, PAAm-hyaluronan, PAAm-chitosan, PEGDA-alginate and PEGDA-hyaluronan (note: PAAm stands for polyacrylamide and PEGDA stands for polyethylene glycol diacrylate)^20^. We further explore a number of applications taking advantage of the robust and microstructured hydrogel–elastomer hybrids including anti-dehydration tough hydrogel with elastomeric coating, stretchable diffusive and reactive hydrogel–elastomer microfluidics, and stretchable hydrogel circuit board patterned on elastomer. The current study not only addresses the long-lasting challenge of developing robust hydrogel–elastomer hybrids, but also makes new applications in various fields possible by introducing a new way to harness distinctive yet complementary advantages of hydrogels and elastomers.

## Results

### Fabrication of hydrogel–elastomer hybrids

**Figure 1:**
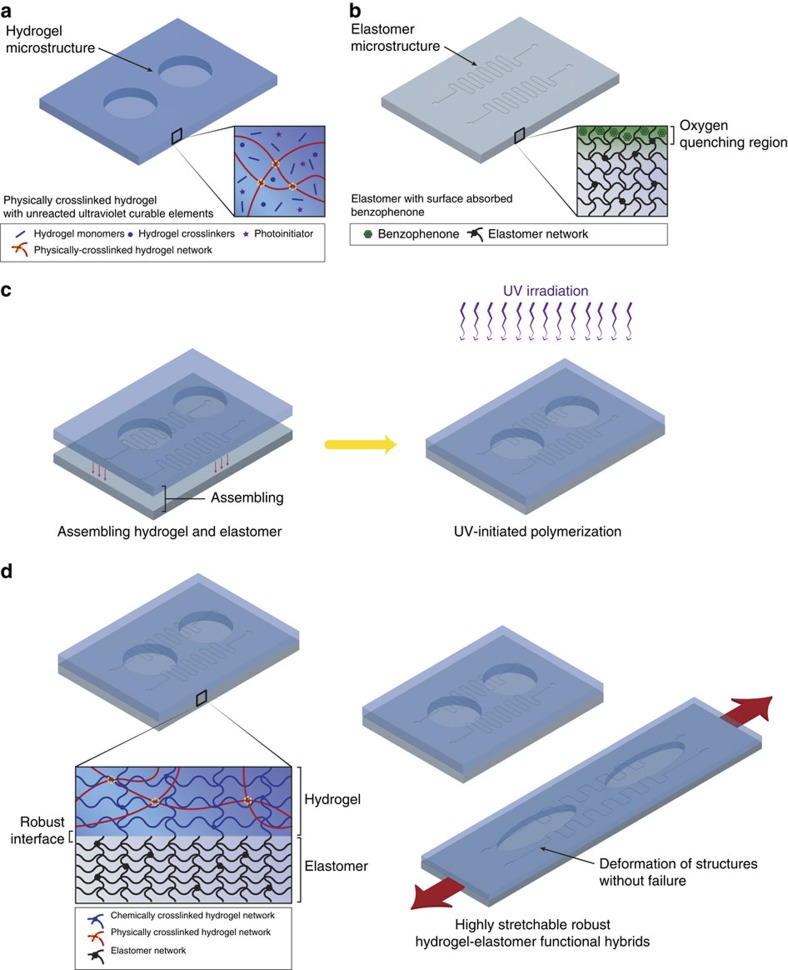
Schematic illustration of the fabrication of robust microstructured hydrogel–elastomer hybrids. (**a**) The hybrids are formed by bonding tough hydrogels of interpenetrating polymer networks with elastomers. One polymer network of the hydrogel is first physically crosslinked, while infiltrated with monomer/macromonomer solution of the other polymer network. The physical crosslinking sets the shape and microstructures of the hydrogel. (**b**) The surface of a cured elastomer with patterned microstructures is treated with benzophenone. (**c**) The pre-shaped hydrogel and elastomer are assembled together followed by ultraviolet irradiation to chemically crosslink the other polymer network in the hydrogel. (**d**) After ultraviolet irradiation, the resultant hydrogel–elastomer hybrid forms extremely robust interfaces due to the covalently anchored polymer network in the hydrogel on elastomer surface. The pre-patterned microstructures in elastomers and hydrogels are also preserved in the hybrid. The hybrids can be highly stretched without interfacial failure.

Although hydrogels and elastomers have been widely used in diverse technologies^1^,^2^,^3^,^4^,^5^,^6^,^7^,^8^,^9^,^10^,^11^,^12^,^13^,^14^,^15^,^16^,^17^,^18^,^19^,^20^,^21^, they still cannot be integrated into hybrid structures with robust interfaces and functional microstructures, mainly due to challenges including elastomer surfaces' inhibition of polymer crosslinking and grafting, and fluidic characters of pre-gel solutions and/or pre-elastomer resins that diminish interfacial microstructures during the formation of hybrids. To address these challenges, we propose a simple yet versatile method to assemble pre-shaped elastomers and hydrogels into hybrids with robust interfaces and functional microstructures (Fig. 1a–c).

The essential ideas of the method are briefly described as follows (see the ‘Methods' section for detailed procedures). Robust hydrogel–elastomer interfaces first require high toughness of the constituent hydrogels^23^. As tough hydrogels generally consist of stretchy polymer networks and other components that dissipate mechanical energy under deformation^27^,^28^,^29^, we interpenetrate covalently crosslinked stretchy polymer networks and physically crosslinked dissipative polymer networks to form tough hydrogels used in the current study^28^,^29^,^30^,^31^. We first physically crosslink the dissipative network to form a hydrogel infiltrated with monomer/macromonomer solution of the stretchy network, which can be crosslinked in future steps (Fig. 1a). The physical crosslinking allows the hydrogel to maintain its pre-designed shapes and microstructures during assembly with elastomers. Similarly, the elastomer can also be cured with predetermined shapes and microstructures before bonding with hydrogels. To address elastomers' oxygen inhibition effect, we treat elastomer surfaces with 10 wt.% benzophenone in ethanol solution via swelling-driven surface absorption of benzophenone solution (Fig. 1b)^32^,^33^. The benzophenone also acts as a ultraviolet-assisted grafting agent for covalently crosslinking hydrogel polymers on elastomer surfaces^32^,^33^,^34^,^35^,^36^,^37^,^38^,^39^,^40^ (Supplementary Fig. 1). Thereafter, the pre-shaped hydrogel and elastomer are assembled into a hybrid, and the stretchy polymer network in the hydrogel is then crosslinked and grafted on the surface of elastomer—leading to robust and microstructured interfaces capable of large deformation (Fig. 1c,d). Furthermore, since the proposed method does not rely on specific types of polymers, it is widely applicable to various commonly used elastomers including polydimethylsiloxane Sylgard 184, polyurethane, latex, VHB and Ecoflex and tough hydrogels including PAAm-algiante, PAAm-hyaluronan, PAAm-chitosan, PEGDA-alginate and PEGDA-hyaluronan^20^.

### Robustness of hydrogel–elastomer hybrids

**Figure 2:**
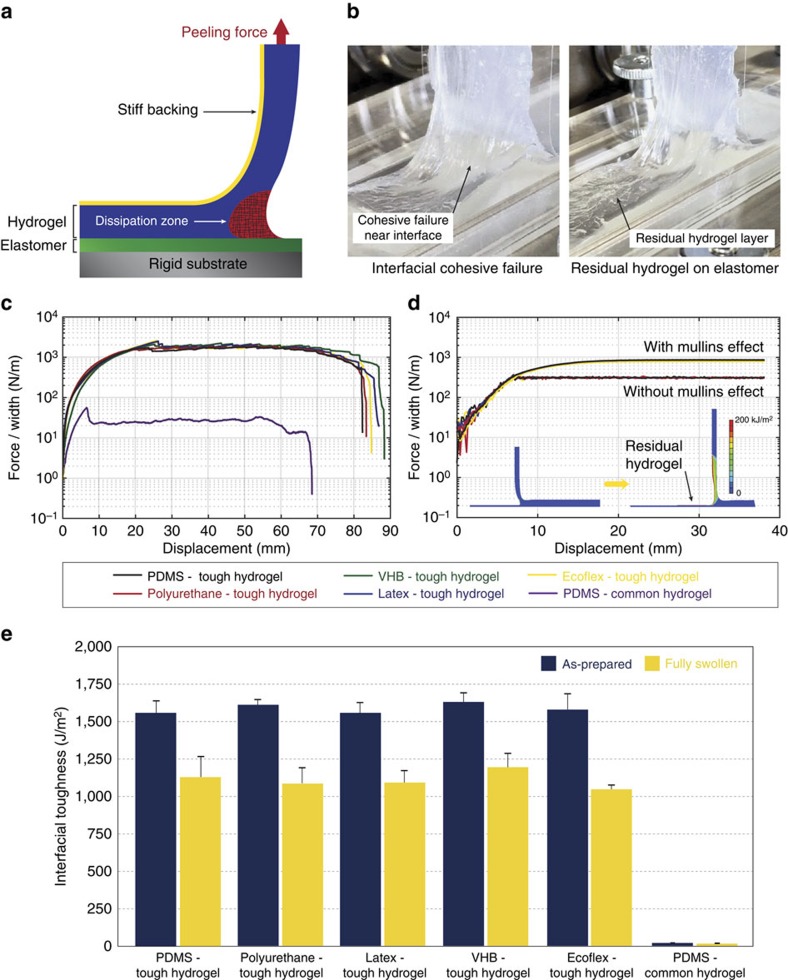
Experimental and simulation results of 90°-peeling tests on hydrogel–elastomer hybrids. (**a**) Schematic illustration of the 90°-peeling test (ASTM D 2861) on various hydrogel–elastomer hybrids. A stiff backing is introduced to prevent elongation of hydrogel sheet along the peeling direction. (**b**) Photos of the hydrogel–elastomer interface during peeling test. The tough hydrogel undergoes a cohesive failure during the peeling test, leaving a thin residual layer of hydrogel ∼0.2 mm on the elastomer substrates. (**c**) The measured peeling forces per width of the hydrogel sheets for various hydrogel–elastomer hybrids (in as-prepared state). (**d**) The calculated peeling forces per width of the hydrogel sheets for various hydrogel–elastomer hybrids in finite-element simulation. The simulated interfacial toughness is significantly decreased as dissipative properties is eliminated in the hydrogel (that is, without Mullins effect) while maintaining other parameters the same. Note that inset pictures are snapshots of the 90°-peeling simulation. The contours indicate the energy dissipation per unit area in the material. (**e**) Summary of measured interfacial toughness of various hydrogel–elastomer hybrids using the proposed method at both as-prepared and fully swollen states. Values in **e** represent mean and the error bars represent the s.d. of measured interfacial toughness for each elastomer materials (*n*=3–5).

To quantify the robustness of hydrogel–elastomer hybrids fabricated with the proposed method, we first use the standard 90°-peeling test to measure interfacial toughness of hydrogel sheets (thickness, 3 mm) bonded on elastomer substrates (thickness, 1 mm) as illustrated Fig. 2a (see the ‘Methods' section for details). The bottom surface of elastomer is constrained on a thick rigid plate during the peeling test; while the top surface of the hydrogel is attached to a thin stiff backing (polyethylene terephthalate (PETE) film of ∼70 μm thickness), which prevents the hydrogel's elongation along the peeling direction^41^. Therefore, the measured steady-state peeling force per unit width of the hydrogel sheet gives the interfacial toughness of the hybrid.

As shown in Fig. 2c,e, the proposed method is indeed capable of achieving consistently high interfacial toughness for PAAm-alginate tough hydrogels (in as-prepared state) bonded onto various elastomers including polydimethylsiloxane Sylgard 184 (1,560 Jm^−2^), polyurethane (1,610 Jm^−2^), latex (1,520 Jm^−2^), VHB (1,630 Jm^−2^) and Ecoflex(1,580 Jm^−2^). Notably, the measured interfacial toughness for PAAm-alginate hydrogel on different elastomers are very similar to one other (Fig. 2c,e). This similarity can be explained by the images of the hydrogel–elastomer interface during peeling test (Fig. 2b). It can be seen that the tough hydrogel, instead of the hydrogel–elastomer interface, undergoes a cohesive failure near the interface during the peeling test—leaving a residual layer of hydrogel (∼0.2 mm thickness) on the elastomer substrates. To better understand the adhesion between tough hydrogels and elastomers, we further vary the grafting density of PAAm in PAAm-alginate tough hydrogels on surfaces of polydimethylsiloxane Sylgard 184 by changing the concentration of benzophenone in the surface treatment solution while maintaining the same treatment time. As the benzophenone concentration in the surface treatment solution increases from 2 wt.% to 8 wt.%, the measured interfacial toughness increases from 200 to 900 Jm^−2^ (Supplementary Fig. 2b,c). This trend is consistent with previous observations on the increase of interfacial toughness between elastomers with area density of polymer chains that connect the elastomers^42^. In addition, when the benzophenone concentration is in the range of 2∼8 wt.%, the samples undergo adhesive failure without leaving an obvious residual hydrogel layer during the peeling test as shown in Supplementary Fig. 2a. On the other hand, when the benzophenone concentration in the surface treatment solution rises above 9 wt.%, the measured interfacial toughness remains approximately the same (∼1,500 Jm^−2^), and cohesive failures of the hydrogels occur consistently. These results indicate that the grafting density of hydrogel polymers on the surface of elastomer can strongly influence both the mode of failure (that is, adhesive or cohesive failure) and the measured interfacial toughness.

In addition, as shown in Supplementary Fig. 3b, the proposed method can also enable robust bonding between various other tough hydrogels including PAAm-hyaluronan (821 Jm^−2^), PAAm-chitosan (436 Jm^−2^), PEGDA-alginate (427 Jm^−2^) and PEGDA-hyaluronan (262 Jm^−2^) and elastomers (for example, polydimethylsiloxane Sylgard 184), demonstrating the versatility of the proposed method. Since 10 wt.% benzophenone solution is used to treat the elastomer surfaces in these samples, the tough hydrogels also fail cohesively during the peeling tests. Therefore, the interfacial toughness of these hydrogel–elastomer hybrids measured with the peeling test is strongly correlated with the fracture toughness of the hydrogels measured with the pure-shear test^23^,^43^ (Supplementary Fig. 3c). These results indicate that the measured interfacial toughness of the hydrogel–elastomer hybrid is limited by the toughness of the hydrogel if the cohesive failure of the hydrogel occurs during the test.

As hydrogel–elastomer hybrids can be used in wet environments, we further carry out the 90°-peeling test on fully swollen hydrogel–elastomer hybrids by immersing them in deionized water for over 24 h until they reach equilibrium swollen state. As shown in Fig. 2e and Supplementary Fig. 4, The measured interfacial toughness of the fully swollen samples are still consistently high and similar to one another, that is, polydimethylsiloxane Sylgard 184 (1,131 Jm^−2^), polyurethane (1,087 Jm^−2^), latex (1,092 Jm^−2^), VHB (1,191 Jm^−2^) and Ecoflex(1,044 Jm^−2^), owing to cohesive failures of the hydrogels during the peeling tests. These results demonstrate that the interfaces of hydrogel–elastomer hybrids prepared with the proposed method are robust in both as-prepared and fully swollen states.

To validate that high toughness of the hydrogel is critical for achieving robust hydrogel–elastomer interface, we bond a common PAAm hydrogel with similar shear moduli as the PAAm-alginate hydrogel (∼30 kPa) on elastomer substrate using a similar method (see the ‘Methods' section for details). While cohesive failure also occurs in the PAAm hydrogels during peeling test, the measured interfacial toughness is 24 and 21 Jm^−2^ for as-prepared and fully swollen samples, respectively—much lower than the values for PAAm-alginate tough hydrogels due to the low fracture toughness of PAAm hydrogels (Fig. 2e)^23^. These results validate that the dissipative properties and high toughness of the hydrogels is critical to achieving robust hydrogel–elastomer hybrids.

To study the effects of benzophenone treatment of elastomer surfaces on hydrogel–elastomer interfaces, we bond common PAAm hydrogel and tough PAAm-alginate hydrogel on elastomer substrates untreated by benzophenone. From Supplementary Fig. 5a, it can be seen that the failure occurs at the hydrogel–elastomer interfaces and the measured interfacial toughness is very low, 2.6 and 3.5 Jm^−2^ for PAAm and PAAm-alginate hydrogels, respectively (Supplementary Fig. 5b,c). These results indicate that the untreated elastomer surfaces indeed hamper the grafting and crosslinking of acrylamide to the surface, leading to very weak hydrogel–elastomer interfaces.

To quantitatively understand the measured interfacial toughness of hydrogel–elastomer hybrids using the proposed method, we use a finite-element model to simulate the 90°-peeling experiment on hydrogel–elastomer hybrids^23^,^43^ (see the ‘Methods' section and Supplementary Fig. 6 for details of the model; Fig. 2d and Supplementary Movie 1). Following experimental observations, we assume that the hydrogel undergoes cohesive failure in the simulation of peeling tests, leaving a residual layer of hydrogel with thickness of 0.2 mm on the elastomer substrate. The interface between the residual layer and the other part of the hydrogel is characterized as a layer of cohesive elements that prescribe the intrinsic fracture energy of the tough PAAm-alginate hydrogel to be 300 Jm^−2^ (ref. 43). The mechanical properties of the tough hydrogel are prescribed by the Ogden model with parameters obtained from mechanical tests on the PAAm-alginate hydrogel (Supplementary Fig. 7)^30^,^44^. The dissipative properties of the hydrogels are characterized by Mullins effect^23^,^43^. From Fig. 2d, it can be seen that different elastomer substrates indeed have negligible effect on the calculated interfacial toughness of the hybrids (that is, ∼900 Jm^−2^), due to cohesive failure of the hydrogels. On the other hand, the simulated interfacial toughness is significantly decreased to the prescribed intrinsic fracture energy of the tough hydrogel (that is, 300 Jm^−2^), if we eliminate dissipative properties (that is, eliminate Mullins effect) in the hydrogel while maintaining other parameters the same. It should be noted that the intrinsic fracture energy of the tough PAAm-alginate hydrogel (that is, ∼300 Jm^−2^) is still much higher than the fracture toughness of common PAAm hydrogel with similar modulus (that is, ∼24 Jm^−2^). These results validate the importance of high fracture toughness and dissipative properties of the hydrogels in achieving robust interfaces.

**Figure 3:**
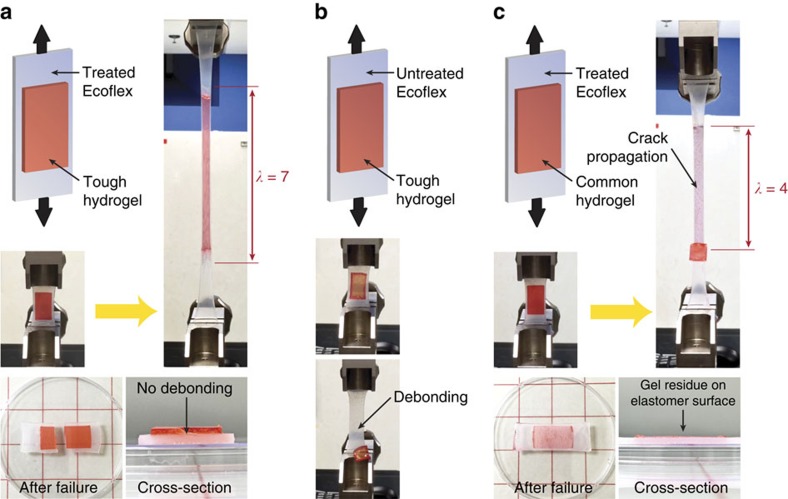
Hydrogel–elastomer hybrids under uniaxial stretches. (**a**) PAAm-alginate hydrogel bonded on Ecoflex elastomer using the proposed method can withstand large deformation (stretch ∼7) without debonding. The robust hydrogel–elastomer bonding is intact even after fracture of the hybrid. (**b**) PAAm-alginate hydrogel bonded on Ecoflex elastomer untreated by benzophenone detaches from the elastomer under small deformation (that is, stretch ∼1.1) due to weak adhesion. (**c**) PAAm hydrogel bonded on Ecoflex elastomer using the proposed method fails under large deformation (stretch ∼4) due to crack propagation in the brittle bulk PAAm hydrogel. Note that red food dyes are added into the hydrogels to enhance the contrast between hydrogels and elastomers.

In addition to the peeling test, the high robustness of hydrogel–elastomer hybrids fabricated with the proposed method can also be demonstrated in other modes of deformation. For example, a laminate of PAAm-alginate hydrogel bonded on Ecoflex sheet can be stretched up to seven times of its original length without delamination (Fig. 3a and Supplementary Movie 2). The laminate is fractured under higher stretch (that is, stretch ∼7.1), but the hydrogel–elastomer interface remains intact without debonding (Fig. 3a). In contrast, the PAAm-alginate hydrogel adhered on Ecoflex elastomer untreated by benzophenone detaches from the elastomer under very small deformation (that is, stretch ∼1.1; Fig. 3b and Supplementary Movie 3)–demonstrating the critical role of benzophenone in achieving robust hydrogel–elastomer interfaces. Interestingly, common PAAm hydrogel bonded on elastomers treated by benzophenone using a similar method (Supplementary Fig. 8) can also sustain a relatively high stretch up to four times until crack propagation within the brittle bulk hydrogel (Fig. 3c and Supplementary Movie 4).

### Applications of robust hydrogel–elastomer hybrids

**Figure 4:**
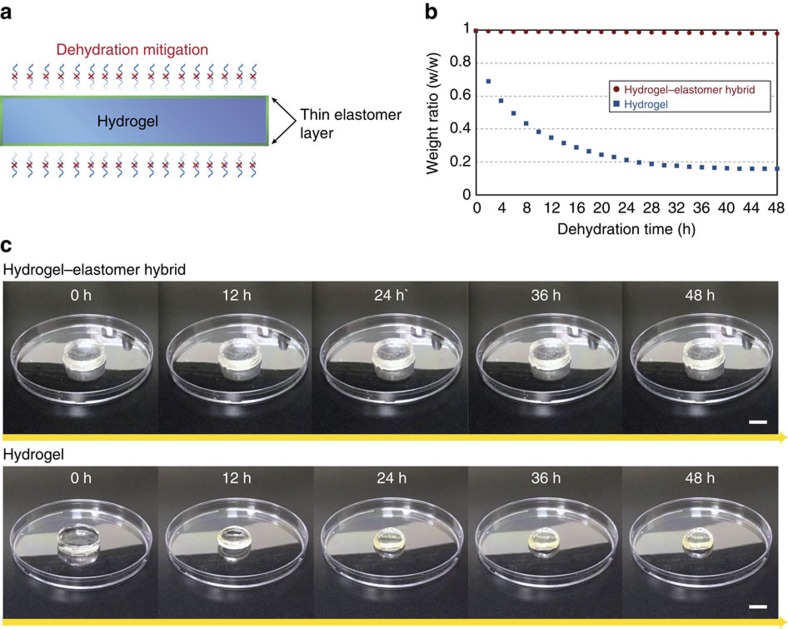
Anti-dehydration hydrogel–elastomer hybrid. (**a**) Schematic illustration of the anti-dehydration elastomeric coating for hydrogels. A very thin layer of Ecoflex elastomer robustly bonded to the hydrogel can effectively prevent evaporation of water from the hydrogel. (**b**) The hydrogel–elastomer hybrid does not show noticeable change in its weight under the ambient testing conditions (24 °C and 50% humidity) for 48 h; whereas hydrogel without elastomeric coating loses most of its water content after 48 h. (**c**) Snapshots of the hydrogel–elastomer hybrid and hydrogel during the dehydration experiments. Scale bar, 10 mm (**c**).

The robust hydrogel–elastomer hybrids enable us to explore various applications otherwise unachievable with hydrogel or elastomer systems alone. For instance, with recent developments of hydrogel-based devices and machines, the dehydration of hydrogels in dry environments becomes a critical challenge in the field^45^. On the other hand, the elastomer-like epidermis in mammalian skin can effectively prevent the hydrogel-like body it covers from dehydration. Inspired by the function of epidermis, we propose to use thin elastomer films robustly bonded on hydrogels to form anti-dehydration hydrogel–elastomer hybrids (Fig. 4). To test the hypothesis, we apply very thin (∼100 μm) Ecoflex coatings on a PAAm-alginate hydrogel disk (25 mm diameter and 6 mm of thickness) using the proposed fabrication method (Fig. 4a). For Ecoflex with thickness in the range of 100–300 μm, the trans-membrane water loss has been reported to be independent of its thickness and as low as 1.5 gh^−1^ m^−2^ (refs 46, 47). Thereafter, we carry out dehydration tests on the hydrogel–elastomer hybrid and an uncoated PAAm-alginate hydrogel with the same dimensions under the ambient conditions (24 °C and 50% humidity) for 48 h (Fig. 4c). We find that the hydrogel–Ecoflex hybrid does not exhibit noticeable change in its weight over 48 h, while the uncoated hydrogel loses its weight close to its original water contents (∼85 wt.%) after 48 h, demonstrating the effective anti-dehydration of the hydrogel–elastomer hybrids (Fig. 4b,c and Supplementary Movie 5). In addition, we find that the Ecoflex coating does not significantly affect the overall mechanical properties of the bulk hydrogel, owing to the much lower thickness (100 μm) than the hydrogel, low modulus (∼30 kPa) and high stretchability (∼7 times) of the Ecoflex coating (Supplementary Fig. 9).

**Figure 5:**
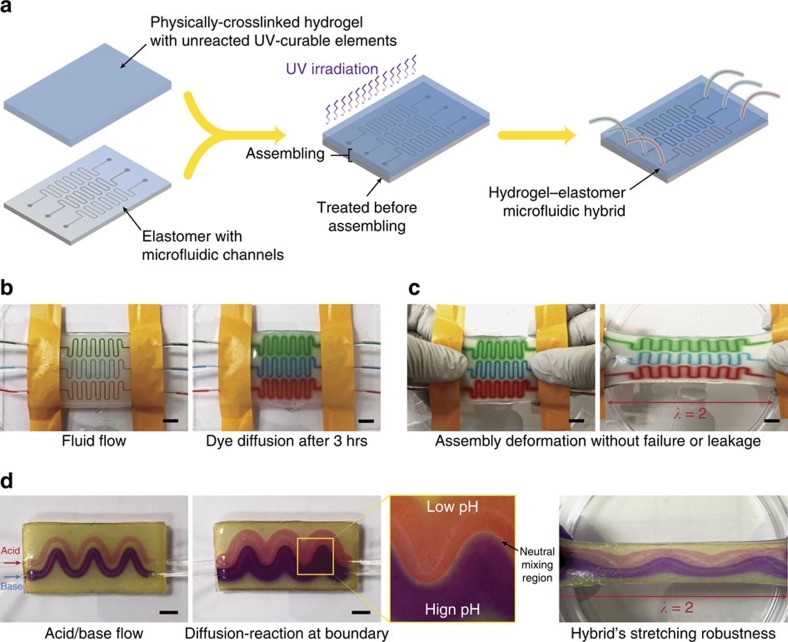
Stretchable diffusive and reactive microfluidic chips based on hydrogel–elastomer hybrids. (**a**) Schematic illustration of the fabrication procedure for hydrogel–elastomer microfluidic chip. (**b**) The resultant hydrogel–elastomer microfluidic hybrid supports convection of chemicals (represented by food dye in different colours) in the microfluidic channels and diffusion of chemicals in the hydrogel. (**c**) The hydrogel–elastomer microfluidic hybrid can maintain functionality under large deformation (for example, stretch ∼2) without debonding failure or leakage thanks to the robust interfacial bonding. (**d**) The hydrogel–elastomer microfluidic hybrid can be used as a platform for diffusion-reaction study. Acid (pH ∼3) and base (pH ∼10) solutions from two microfluidic channels diffuse in the pH-sensitive hydrogel and form regions of different colours (light red for acid and dark violet for base). The reaction of acid and base solutions in the hydrogel further form a neutral region (pH ∼7, light green colour). Scale bars, 10 mm (**b**–**d**).

In mammalian skin, the blood and lymphatic vessels in the hydrogel-like dermis provide functions of nourishment and waste removal through a combination of convention and diffusion. Inspired by the functions of micro-vessels in skin, we propose to develop robust yet flexible hydrogel–elastomer hybrids with micro-channels patterned on interfaces to enable simultaneous convection, diffusion and reaction of different species in the hybrids under large deformation (Fig. 5). Following the proposed method, we assemble diffusive PAAm-alginate hydrogel sheet and flexible Ecoflex elastomer with microfluidic channels into robust integrated hybrids (Fig. 5a). The resultant robust and highly stretchable hybrid works as a functional microfluidic assembly with unique features such as convection of solvents in the microfluidic channels plus diffusion through the hydrogel part (Fig. 5b and Supplementary Movie 6). Notably, the robust interfacial bonding achieved by the proposed method enables large deformation of the hydrogel–elastomer hybrid without failure or debonding-driven leakage of solvents (Fig. 5c and Supplementary Movie 6). In addition, we carry out diffusion-reaction test through wavy microfluidic channels on Ecoflex assembled with PAAm-alginate hydrogel that contains pH-indicating molecules (see the ‘Methods' section for details on fabrication) (Fig. 5d). Acid (pH ∼3) and base (pH ∼10) solutions from two microfluidic channels can diffuse in the pH-sensitive hydrogel and form regions of different colours (light red indicating acid and dark violet indicating base). The reaction of acid and base solutions further forms a neutral region in the hydrogel (pH ∼7, light green colour; Fig. 5d and Supplementary Movie 7).

**Figure 6:**
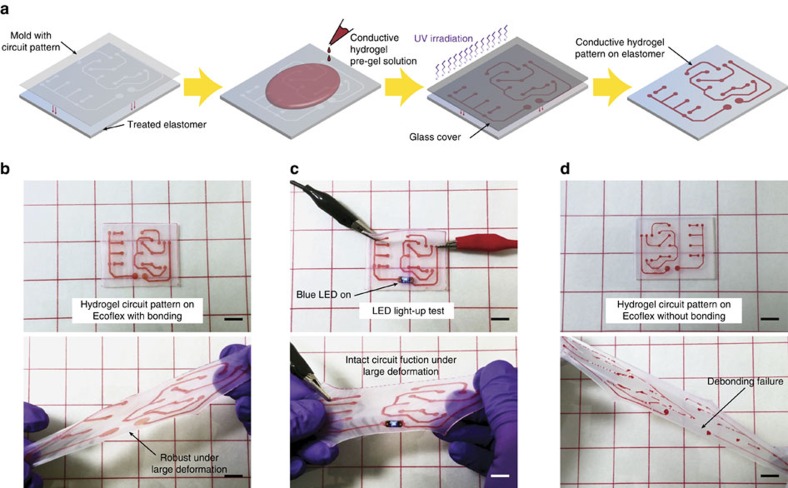
Stretchable hydrogel circuit board patterned on elastomer. (**a**) Schematic illustration of fabrication procedure for conductive hydrogel circuit patterned on flexible elastomer substrate. (**b**) Ionically conductive PAAm-alginate hydrogel circuit bonded on an Ecoflex elastomer substrate using the proposed method is robust under large deformation without visible failure. (**c**) The hydrogel circuit board connected with an AC power source can light up LED, and it can maintain its electrical functionality even under severe deformation. (**d**) A hydrogel circuit bonded on Ecoflex elastomer without benzophenone treatment delaminates and fails under deformation, due to the weak hydrogel–elastomer bonding. Scale bars, 10 mm (**b**–**d**).

As another example, we demonstrate a robust and conductive hydrogel circuit on flexible elastomer substrate (Fig. 6). Conductive hydrogels have been used in transparent electroactive speaker^17^, sensors^18^ and electrical signal transmission^19^, but unreliable integration of conductive hydrogels to elastomers greatly limits their applications and reliability. To address the challenge, we utilize the proposed fabrication method to form a robust ionically conductive hydrogel circuit patterned on top of thin Ecoflex substrate that mimics printed circuit board for standard electronics (Fig. 6a). Figure 6b and Supplementary Movie 8 show that the hydrogel circuit fabricated by the proposed method can sustain large deformation and high stretch without noticeable failure. In Fig. 6c and Supplementary Movie 8, we demonstrate the functionality of the fabricated hydrogel circuit board by lighting up LED with an a.c. power source connected to the hydrogel circuit. The conductive hydrogel circuit can indeed maintain its electrical functionality even under severe deformation (Fig. 6c and Supplementary Movie 8). The electrical resistance of the conductive hydrogel patterned on Ecoflex substrate remains almost the same after 100 cycles of stretch to 3.5 times. In addition, the relation between electrical resistance and stretch follows *R/R*_*0*_=λ^2^, where *R*_*0*_ is the resistance before deformation and *R* is the resistance after stretch of λ from the initial state^17^,^19^ (Supplementary Fig. 10b). To validate the importance of robust hydrogel–elastomer interfaces for such hydrogel circuit, we further perform a control test by deforming the same ionically conductive hydrogel circuit patterned on elastomer substrate without benzophenone treatment (that is, weak interfaces; Fig. 6d). The hydrogel pattern without robust interface easily debonds from the elastomer substrate and fails under deformation, indicating the importance of robust bonding of hydrogel on elastomer substrates for the stretchable hydrogel circuits (Fig. 6d).

## Discussion

Natural hybrids of hydrogel-like dermis and elastomer-like epidermis in mammalian skins possess robust interfaces and functional microstructures that have not been achieved in synthetic hydrogel–elastomer systems. Here, we report a simple yet versatile method to create synthetic hydrogel–elastomer hybrids with interfacial bonding tougher than epidermis–dermis interfaces and functional micro-channels and micro-patterns inspired by blood and lymphatic vessels in mammalian skins. The method integrates three innovations in fabrication of soft hybrids. First, pre-shaping both elastomers and hydrogels before bonding to conserve their microstructures. Second, modification of cured elastomer surfaces with benzophenone for chemical bonding with hydrogels. Third, harnessing dissipative properties of tough hydrogels to achieve robust interfaces. The method is widely applicable to various commonly used elastomers including polydimethylsiloxane Sylgard 184, polyurethane, latex, VHB and Ecoflex and tough hydrogels including PAAm-alginate, PAAm-hyaluronan, PAAm-chitosan, PEGDA-alginate and PEGDA-hyaluronan. The robust hydrogel–elastomer hybrids allow us to harness distinctive but complementary advantages of both elastomers and hydrogels and explore diverse applications including anti-dehydration hydrogels, stretchable diffusive and reactive microfluidic chips, and stretchable hydrogel circuit board.

In the current study, we demonstrate the fabrication of hydrogel–elastomer hybrids with dimensions up to a few centimeters. The facileness and versatility of the proposed method makes it suitable for large-scale manufacturing and potential incorporation into advanced fabrication techniques such as additive manufacturing for both elastomers and hydrogels^31^,^48^,^49^. In addition, the ability to fabricate extremely robust and microstructured hydrogel–elastomer hybrids makes a number of future research directions and applications possible. For example, elastomer-based flexible electronic devices integrated with hydrogels may lead to development of a new class of flexible bio-electronic devices for seamless interfacing between human body and engineering devices^20^. Biocompatible and/or biodegradable hydrogels containing living organisms (for example, bacteria and cells) integrated with existing elastomer-based devices may be a promising route toward more creative utilization of living organisms for engineering applications^31^,^48^,^50^,^51^,^52^. Microfluidic systems based on hydrogel–elastomer hybrids may provide more efficient platforms for diverse biomedical studies owing to its unique integration of convection, diffusion, reaction and deformation^6^.

## Methods

### Materials

Unless otherwise specified, the chemicals used in the current work were purchased from Sigma-Aldrich and used without further purification. For the covalently crosslinked stretchy polymer networks in the tough hydrogels, acrylamide (AAm; Sigma-Aldrich A8887) was the monomer used for the PAAm networks, and 20 kDa PEGDA (Sigma-Aldrich 767549) was the macromonomer used for the PEGDA networks. For the PAAm hydrogel, *N,N*-methylenebisacrylamide (MBAA; Sigma-Aldrich 146072) was used as crosslinker and 2-Hydroxy-4′-(2-hydroxyethoxy)-2-methylpropiophenone (Irgacure 2959; Sigma-Aldrich 410896) was used as photoinitiator. For the PEGDA hydrogel, Irgacure 2959 was used as photoinitiator. For the physically crosslinked dissipative polymer networks in the tough hydrogels, a number of ionically crosslinkable biopolymers were used including sodium alginate (Sigma-Aldrich A2033) ionically crosslinked with calcium sulfate (Sigma-Alginate C3771), chitosan (Sigma-Aldrich 448869) ionically crosslinked with sodium tripolyphosphate (Sigma-Aldrich 238503) and sodium hyaluronan (Sigma-Aldrich H5542) ionically crosslinked with iron chloride (Sigma-Aldrich 157740). For elastomer surface treatment, benzophenone (Sigma-Aldrich B9300) was used. To visualize pH change within the hydrogel–elastomer microfluidic chip, universal pH indicator solution (Sigma-Aldrich 36828), hydrogen chloride (Sigma-Aldrich 38280) and sodium hydroxide (Sigma-Aldrich 795429) were used. Glucose (Sigma-Aldrich G8270) and glucose oxidase (Sigma-Aldrich G7141) were used as an oxygen scavenger in hydrogels in the tests for the effect of elastomer surface treatments.

For elastomers, Sylgard 184 (polydimethylsiloxane; Dow Corning), Ecoflex (Smooth-On), polyurethane (Smooth-On), latex (McMaster Carr) and VHB (3 M) were used. In the 90°-peeling experiments, borosilicate glass (McMaster Carr) was used as a rigid substrate bonded on the bottom surface of the elastomer. As a stiff backing for the hydrogel sheet, PETE film (70 μm; ePlastics) were used together with cyanoacrylate (Loctite, Henkel). In the conductive hydrogel circuit experiments, sodium chloride (Sigma-Aldrich 746398) solution was used as an electrolyte. For hydrophobic coating of glass moulds and covers, Rain-X (ITW Inc.) solution was used.

### Bonding hydrogels on elastomers

The surfaces of elastomers were treated by absorbing benzophenone. The elastomer surfaces were thoroughly cleaned with methanol and deionized water, and completely dried with nitrogen gas before the benzophenone treatment. Thereafter, benzophenone solution (10 wt.% in ethanol) was applied onto the elastomer to evenly cover the entire elastomer surface for 2 min at room temperature. Then, the elastomer was washed with methanol three times and completely dried with nitrogen gas^33^.

Physically crosslinked hydrogel was prepared by mixing 10 ml of a carefully degassed aqueous pre-gel solution (12.05 wt.% AAm, 1.95 wt.% sodium alginate and 0.017 wt.% MBAA for the PAAm-alginate hydrogel; 18 wt. % AAm, 2 wt. % sodium hyaluronan and 0.026 wt.% MBAA for the PAAm-hyaluronan hydrogel; 24 wt.% AAm, 2 wt.% chitosan and 0.034 wt.% MBAA for the PAAm-chitosan hydrogel; 20 wt.% PEGDA and 2.5 wt.% sodium alginate for the PEGDA-alginate hydrogel; 20 wt.% PEGDA and 2 wt.% sodium hyaluronan for the PEGDA-hyaluronan hydrogel) with ionic crosslinkers (20 × 10^−3^ M concentration of calcium sulfate in the PAAm-alginate hydrogel; 3 × 10^−3^ M concentration of iron chloride in the PAAm-hyaluronan hydrogel; 3 × 10^−3^ M concentration of sodium tripolyphosphate in the PAAm-chitosan hydrogel; 20 × 10^−3 ^M concentration of calcium sulfate in the PEGDA-alginate hydrogel; 3 × 10^−3^ M concentration of iron chloride in the PEGDA-hyaluronan hydrogel) and Irgacure 2959 (0.2 wt.%). The mixture was mixed quickly, poured onto glass mould, and then covered by glass plate with hydrophobic coating. The hydrogel was kept in nitrogen chamber for 1 h to allow the formation of physically crosslinked network. Thereafter, the physically crosslinked hydrogel was gently removed from the mould and assembled with freshly treated elastomer followed by ultraviolet irradiation in a ultraviolet chamber (365 nm ultraviolet; UVP CL-1000) for an hour, during which the PAAm network was covalently crosslinked and bonded onto elastomer surface.

PAAm common hydrogel was prepared by directly curing the degassed pre-gel solution (23 wt.% AAm, 0.051 wt.% MBAA and 0.2 wt.% Irgacure 2959) onto freshly treated elastomer surface inside ultraviolet crosslinker. The crosslinking condition was identical to the PAAm-alginate hydrogel. Note that the shear moduli of the PAAm hydrogel was tuned to match the PAAm-alginate hydrogel's modulus (30 kPa) based on the previously reported data^30^.

### Mechanical testing

All tests were performed in ambient air at room temperature. The hydrogels and hydrogel–elastomer interfaces maintained consistent properties over the time of the tests (that is, approximately a few minutes), during which the effect of dehydration is not significant. The interfacial toughness of various hydrogel–elastomer hybrids was measured using the standard 90°-peeling test (ASTM D 2861) with mechanical testing machine (2 kN or 20 N load cells; Zwick/Roell Z2.5) and 90°-peeling fixture (Test Resources, G50). All elastomer substrates were prepared with 2.5 cm in width, 7.5 cm in length and 1 mm in thickness. polydimethylsiloxane and Ecoflex were adhered on borosilicate glass plate using oxygen plasma treatment (Harrick Plasma PDC-001). Latex and polyurethane were adhered on glass plate by with epoxy adhesives. VHB was simply adhered onto glass plate as it was provided in two-sided tape form. Hydrogels were bonded onto elastomer surfaces following the abovementioned procedure with the size of 100 × 15 × 3 mm (length × width × thickness). As a stiff backing for the hydrogel, PETE film was bonded onto the hydrogel with cyanoacrylate adhesive. The resultant samples were tested with the standard 90°-peeling test with a constant peeling speed of 50 mm min^−1^. The measured peeling force reached a plateau (with slight oscillations), as the peeling process entered steady state. The interfacial toughness Γ was determined by dividing the plateau force *F* by the width of the hydrogel sheet *W*.

To investigate the effect of elastomer surface treatment on interfacial toughness and failure modes of hydrogel bonded on elastomers, the same 90°-peeling test was performed using PAAm-alginate tough hydrogel and polydimethylsiloxane substrate with the same sample size and testing conditions. The surface treatment time for polydimethylsiloxane substrate was fixed to 2 min, while the concentration of benzophenone in the surface treatment solutions was varied from 2 wt.% to 10 wt.%. As PAAm-alginate tough hydrogel cannot be successfully cured on top of polydimethylsiloxane with the surface treatment solution containing <5 wt.% of benzophenone due to the effect of oxygen inhibition, 2 wt.% of glucose and 0.02 wt.% of glucose oxidase were added as an oxygen scavenger into the prescribed PAAm-alginate pre-gel solution.

For uniaxial-tensile tests of hydrogel–elastomer hybrids, PAAm-alginate tough hydrogel and PAAm common hydrogel with size of 50 × 20 × 3 mm (length × width × thickness) were bonded onto Ecoflex substrate following the abovementioned procedure. For physically attached samples, the same size of PAAm-alginate tough hydrogel was simply put onto the Ecoflex substrate without any other treatment. The stretching of hybrids was carried out using the mechanical testing machine (2 kN; Zwick/Roell Z2.5) with grip-to-grip separation speed of 100 mm min^−1^.

### Numerical modelling and simulation of 90°-peeling of hydrogel

We developed a two-dimensional finite-element model to simulate the 90°-peeling test of hydrogels bonded on diverse elastomer substrates (that is, Sylgard 184 polydimethylsiloxane, polyurethane, latex, VHB and Ecoflex). A hydrogel strip with length 80 mm and thickness 3 mm was adhered on an elastomer sheet, where we assume the 0.2 mm thin residual hydrogel layer on the elastomer substrate which is connected to the bulk hydrogel via cohesive element (Supplementary Fig. 6). The deformation of the hydrogel strip was assumed to be under plane-strain condition. The elastic properties and energy dissipation of the hydrogel were modelled as the Ogden hyperelastic material and Mullins effect^44^, respectively. The parameters of the model were obtained from the previous studies on the PAAm-alginate hydrogel^23^,^30^. For the elastic properties, the one-term Ogden model can be expressed as


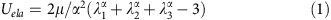


where, *U*_*ela*_ is the strain energy density, *λ*_*i*_ the *i*_th_ principal stretch, *μ* the shear modulus (fitted to be 36.57 kPa), and *α* the Ogden parameter (fitted to be 1.473). The theoretical model for the Mullins effect can be expressed as


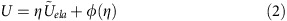



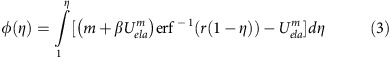



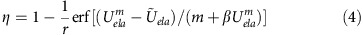


where, *η* is a damage variable (0<*η*≤1), 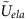 is the strain energy density of perfectly elastic material (that is, the primary loading path is also the unloading path), 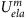 denotes the maximum strain energy density before unloading, the function *φ*(*η*) is referred to as the damage function, erf is the error function, and the material parameters *r*=1.1, *m*=4.076, and *β*=0.2818 were obtained by fitting the model to measured stress–strain hysteresis of the PAAm-alginate hydrogel^30^ (Supplementary Fig. 7). The elastomer substrates were assumed to be elastic materials which were modelled as the Neo–Hookean model, corresponding to an Ogden hyperelastic material with the Ogden parameter of 2 and shear moduli of 0.6 MPa (Sylgard 184 polydimethylsiloxane); 30 kPa (Ecoflex); 1.6 MPa (polyurethane); 1 MPa (latex); 0.6 MPa (VHB).

The stiff backing was modelled as a linear elastic material with very high Young's modulus (that is, 2 GPa) and very low thickness (that is, 100 μm). The cohesive layer on the interface was characterized by a triangular cohesive law with maximum strength *S*_max_ and maximum separation distance *δ*_max_. The damage of the cohesive layer follows the quadratic nominal stress criterion,


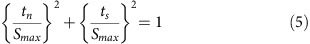


where *t*_*i*_(n,s) represents the nominal stress, and the subscript *n* and *s* indicate deformation normal to and tangential to the interface respectively.

All the numerical simulations were carried out with ABAQUS/Explicit. The hydrogel, stiff backing and elastomer were modelled with CPE4R element, and the cohesive layer at the interface was modelled with COH2D element. The Poisson's ratios of the hydrogel and elastomer were set to be 0.499 to approximate incompressibility. The adhesive interface was uniformly discretized with very fine mesh size (0.1 mm). Mass scaling technique was adopted to maintain a quasi-static process during the peeling simulations. To simulate the peeling test described in the material and experiment section, the left edge of the strip was first rotated 90° and then moved vertically at a constant velocity, with the reaction force on the left edge of the strip recorded. During the simulations, the bottom surface of the elastomer is fixed to mimic the constraint of the rigid glass substrate used in experiments. The interfacial toughness was then calculated as the steady-state reaction force divided by the width of the strip, which is set to be unity in the current model. The parameters for cohesive element between the bulk hydrogel and the residual layer are chosen as *S*_max_ as 200 kPa and *δ*_max_ as 3.0 mm which give the intrinsic fracture energy Γ_0_ of 300 Jm^−2^ for cohesive failure of the hydrogel during peeling test.

### Preparation and testing of anti-dehydration coating

Thin Ecoflex layer was prepared by spin-coating uncured Ecoflex resin on acrylic plate (1,200 rpm for 30 s) with final thickness around 100 μm. The resultant Ecoflex film was placed on inner surfaces of glass mould with the dimension of 25 mm diameter and 6 mm of thickness, and then treated with benzophenone solution following the previously described procedure. Thereafter, the hydrogel–Ecoflex hybrid was prepared by pouring PAAm-alginate hydrogel pre-gel solution into the Ecoflex-covered mould followed by ultraviolet irradiation. Hydrogel without anti-dehydration coating was prepared by crosslinking PAAm-alginate pre-gel solution using the same glass mould without Ecoflex film. The dehydration tests were carried out at room temperature with low humidity (24 °C and 50 % humidity) for 48 h. Weight change of the samples during dehydration tests was recorded every 2 h for comparison.

To test the effect of anti-dehydration elastomeric coating on mechanical property of bulk hydrogel, we prepared a batch of dog-bone-shaped samples of PAAm-alginate hydrogel with 15 mm in width, 35 mm in length and 6.35 mm in thickness. For comparison, another batch of otherwise the same samples were prepared with Ecoflex film (100 μm thickness) robustly bonded on both sides of hydrogels. The tension tests were performed using the mechanical testing machine (2 kN; Zwick/Roell Z2.5) with grip-to-grip separation speed of 50 mm min^−1^.

### Preparation and testing of hydrogel–elastomer microfluidic assembly

Ecoflex microfluidic channel was prepared by curing Ecoflex resin onto silicon wafer mould with predetermined positive SU-8 patterns following conventional soft-lithography techniques^26^. Physically crosslinked PAAm-alginate hydrogel was prepared by the previously described procedure. Physically crosslinked PAAm-algiante hydrogel was gently assembled on top of the Ecoflex microfluidic channel. The assembly was then exposed to ultraviolet irradiation for an hour. To verify microfluidic function and diffusion through hydrogel matrix, 2% aqueous solution of red, blue and green food dyes (McCormick) were supplied through three inlets of microfluidic channels.

The hydrogel–elastomer microfluidic chip for the diffusion-reaction test was prepared following the abovementioned procedures except that 0.1 wt.% of pH indicator solution was added into the PAAm-alginate pre-gel solution. To test the diffusion-reaction of waterborne chemicals, acid solution (0.1 M aqueous hydrogen chloride solution) and base solution (0.1 M aqueous sodium hydroxide solution) were supplied in two microfluidic channels. Note that all hydrogel–elastomer microfluidic chips were kept in humid chamber during tests to avoid dehydration.

### Preparation and testing of conductive hydrogel pattern on elastomer

To form conductive hydrogel circuit board pattern on Ecoflex substrate, thin PETE film (70 μm thickness) with predetermined circuit board pattern was prepared using laser-cutting machine (Epilog Mini/Helix). As a template for hydrogel pattern on elastomer, the film with circuit board pattern was assembled with thin Ecoflex substrate (1 mm thickness) treated with benzophenone solution as previously described. Thereafter, PAAm-alginate pre-gel solution was poured onto the assembly and covered with a glass slide, followed by ultraviolet irradiation for an hour. After ultraviolet irradiation, the glass cover and the PETE film were removed from the Ecoflex substrate leaving robustly bonded PAAm-alginate hydrogel pattern. The hydrogel pattern was made to be ionically conductive by submerging the hybrid in concentrated sodium chloride solution (3 M) for 6 h. To light up a LED on the conductive hydrogel circuit pattern, each ends of pattern were connected to a functional generator (5 V peak-to-peak voltage at 1 kHz).

The electrical property of the conductive hydrogel–elastomer hybrid under deformation was measured using the four-point method^19^. The ionically conductive hydrogel pattern with 50 mm in length, 1 mm in width and 200 μm in thickness was bonded on thin Ecoflex substrate (1 mm thickness) following the abovementioned method. The two ends of the hydrogel pattern were connected in series with a function generator and a galvanometer, and the voltage between two ends were measured with a voltmeter connected in parallel (Supplementary Fig. 10a). The ratio of the measured voltage to the measured current gave the electric resistance of ionically conductive hydrogel pattern. The rate of stretch was kept constant at 100 mm min using a mechanical testing machine. Cyclic extension of the conductive hydrogel–elastomer hybrid was done by mechanical testing machine based on predetermined numbers of cycles.

### Data availability

The data that support the findings of this study are available from the corresponding author on request.

## Additional information

**How to cite this article:** Yuk, H. *et al.* Skin-inspired hydrogel–elastomer hybrids with robust interfaces and functional microstructures. *Nat. Commun.* 7:12028 doi: 10.1038/ncomms12028 (2016).

## Supplementary Material

Supplementary Information Supplementary Figures 1-10 and Supplementary Reference

Supplementary Movie 1 Finite-element simulation of the 90-degree peeling test.

Supplementary Movie 2 Stretching test of PAAm-alginate hydrogel bonded on treated Ecoflex elastomer using the proposed method. Note that the video is edited into 8 times faster than the original speed.

Supplementary Movie 3 Stretching test of PAAm-alginate hydrogel bonded on untreated Ecoflex elastomer. Note that the video is edited into 8 times faster than the original speed.

Supplementary Movie 4 Stretching test of PAAm hydrogel bonded on treated Ecoflex elastomer using the proposed method. Note that the video is edited into 8 times faster than the original speed.

Supplementary Movie 5 Dehydration test of hydrogel-elastomer hybrid and uncoated hydrogel under ambient conditions (24 °C and 50 % humidity).

Supplementary Movie 6 Convection and diffusion of chemicals (represented by food dyes of different colors) in the hydrogel-elastomer microfluidic hybrid. The hybrid maintains its functionality without debonding or leakage under large deformation.

Supplementary Movie 7 Acid (pH ~ 3) and base (pH ~ 10) solutions from two microfluidic channels diffuse in the pH-sensitive hydrogel and form regions of different colors (light red for acid and dark violet for base). The reaction of acid and base solutions in the hydrogel further form a neutral region (pH ~ 7, light green color).

Supplementary Movie 8 Tests of ionically conductive hydrogel circuit patterned on thin Ecoflex elastomer substrate. Robust bonding of hydrogels on elastomers enables the hydrogel circuit to maintain its electrical functionality even under severe deformation.
