# Supplementary material for: Skin-inspired hydrogel–elastomer hybrids with robust interfaces and functional microstructures
Source: Nat Commun. 2016 Jun 27;7:12028. doi: 10.1038/ncomms12028 (PMC4931236; doi:10.1038/ncomms12028)
Supplement: Supplementary Information — Supplementary Figures 1-10 and Supplementary Reference [file ncomms12028-s1.pdf]

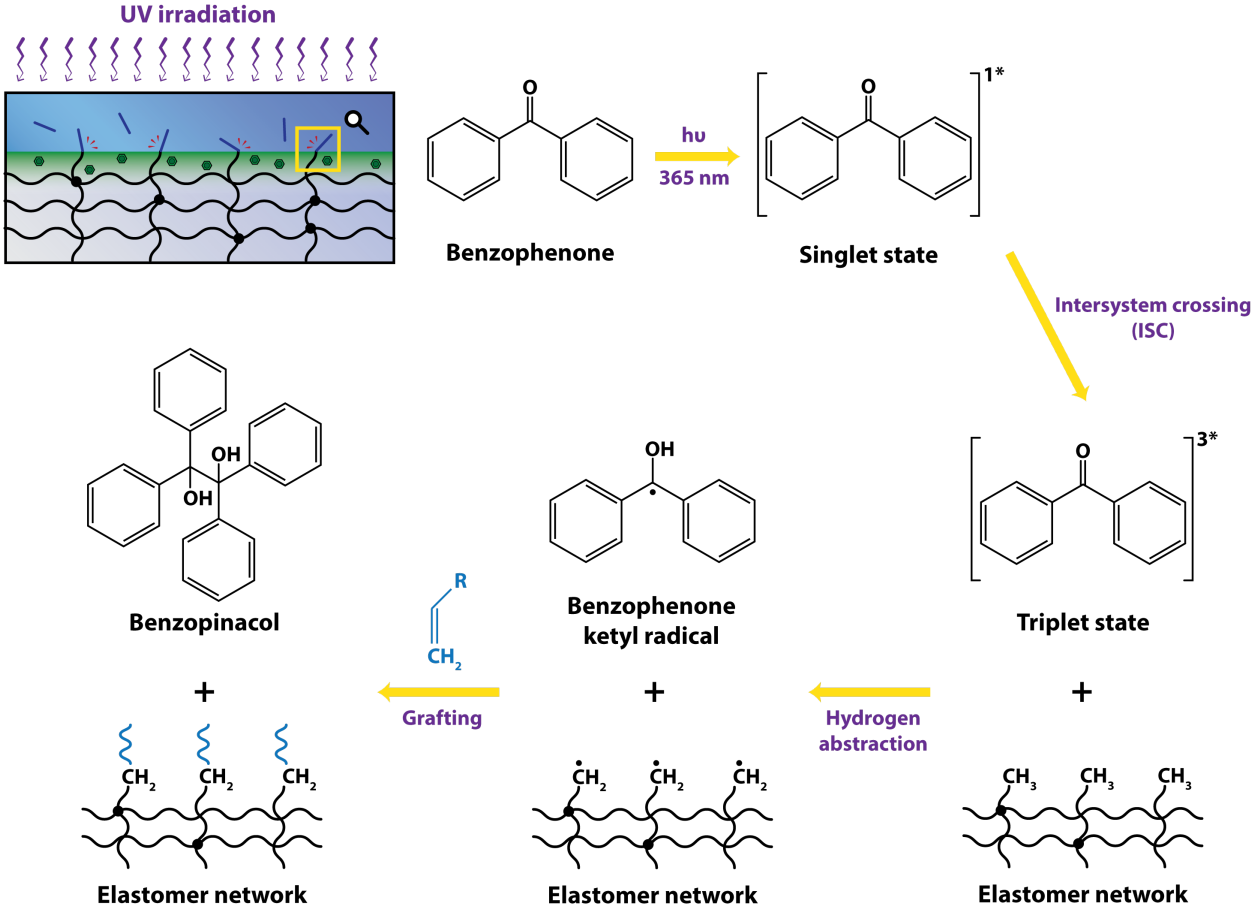

Supplementary Figure 1 | Schematics of benzophenone chemistry for hydrogel bonding. Under UV irradiation, surface absorbed benzophenone is excited into a singlet state which is followed by conversion into a triplet state through intersystem crossing. The triplet-state benzophenone consecutively changes into benzophenone ketyl radical by abstracting a hydrogen from surrounding unreactive C-H bonds in elastomer polymer. Then benzophenone ketyl radical mediates the grafting of stretchy polymer networks of the hydrogel onto the reactive sites on the elastomer surface, generating benzopinacol as a final reaction product.

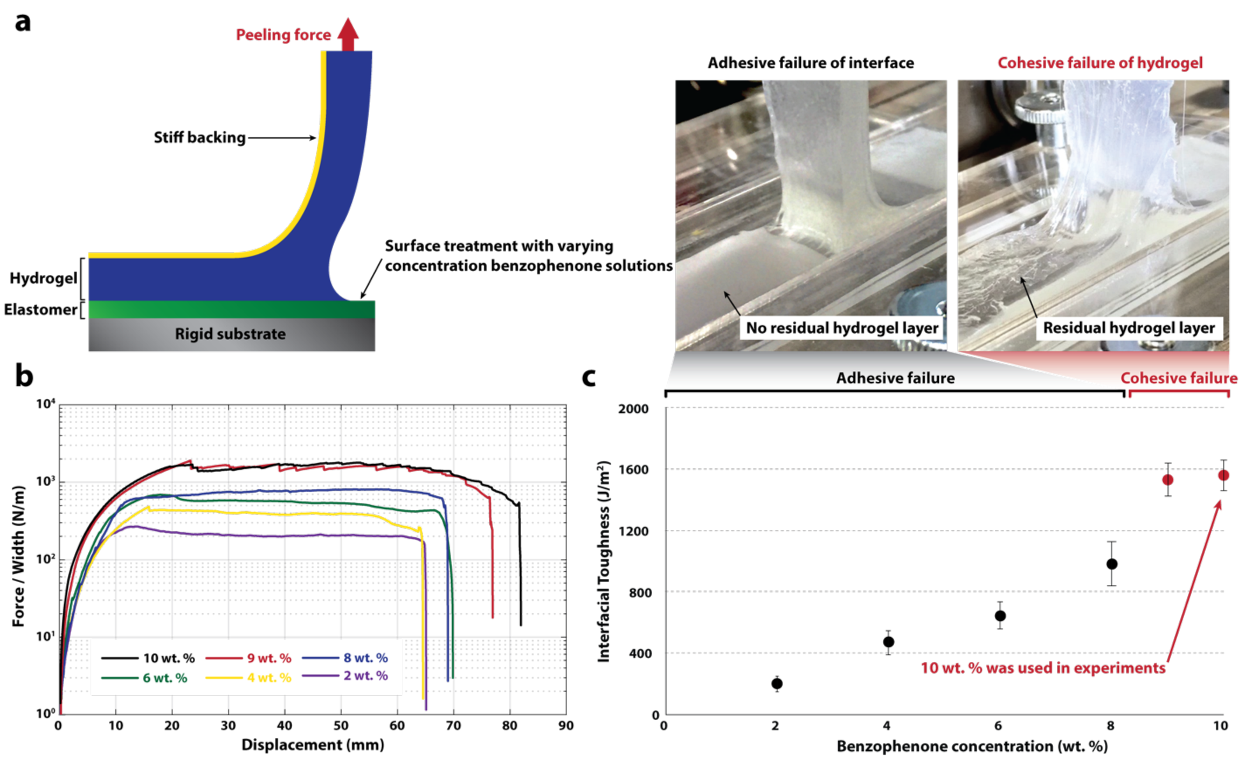

Supplementary Figure 2 | The effect of elastomer surface treatment on interfacial toughness and failure modes of tough hydrogels bonded on elastomers. (**a**) The 90-degree peeling test is used to measure (ASTM D 2861) the interfacial toughness of PAAm-alginate hydrogels bonded on PDMS substrates treated with different concentrations of benzophenone solutions. Photos of the peeling tests show two different modes of interfacial failure (i.e., adhesive failure of the interface and cohesive failure of the hydrogel). (**b**) The measured peeling forces per width of the hydrogel sheets adhered on PDMS for various concentrations of benzophenone used in the surface treatment. (**c**) Summary of the measured interfacial toughness of hydrogel-PDMS hybrids for various concentrations of benzophenone used in elastomer surface treatment. Values in **c** represent mean and the error bars represent standard deviation of the measured interfacial toughness for each concentration (n = 3-5).

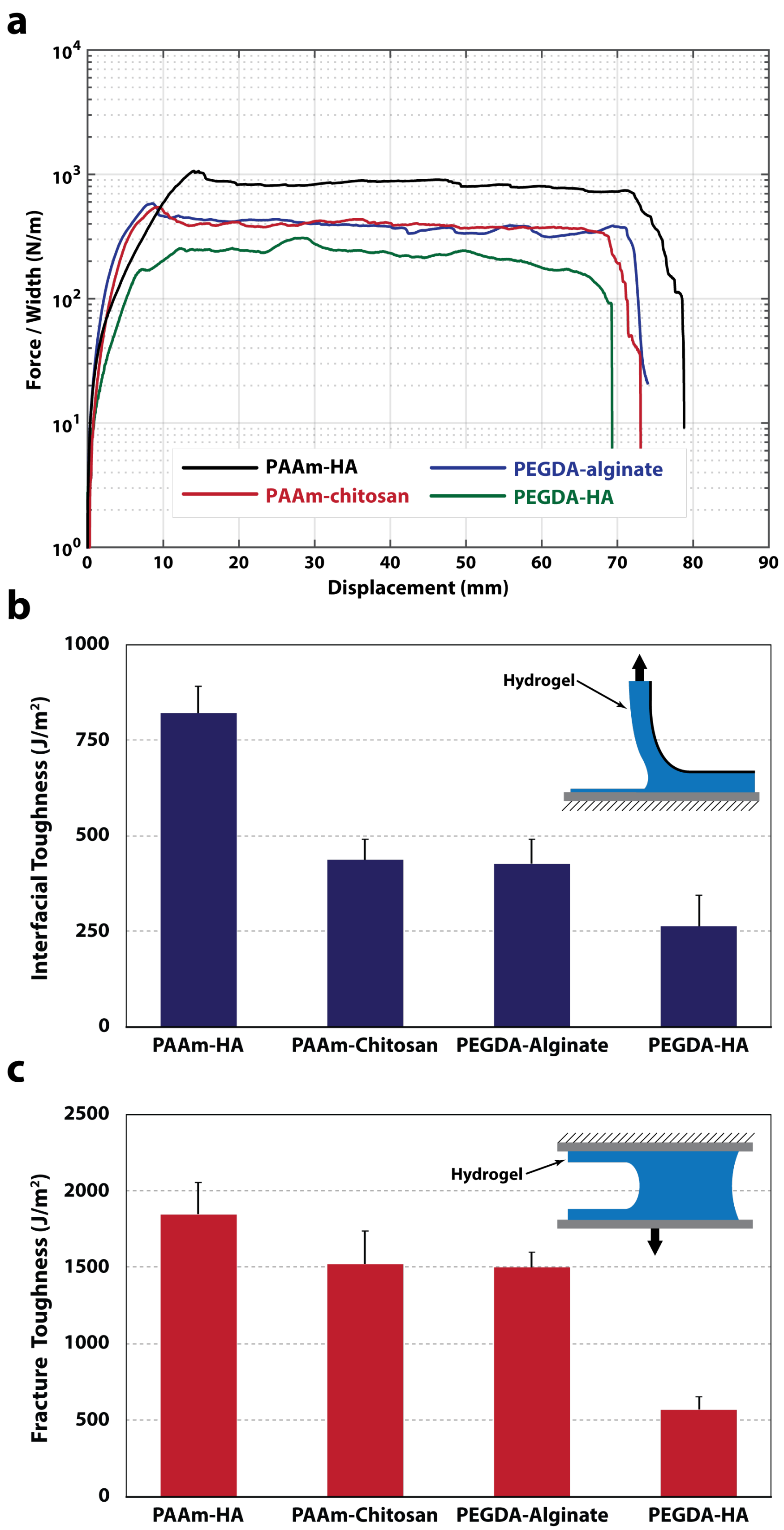

a
Force / Width (N/m)
Displacement (mm)
PAAm-HA
PAAm-chitosan
PEGDA-alginate
PEGDA-HA
b
Interfacial Toughness (J/m²)
Hydrogel
PAAm-HA
PAAm-Chitosan
PEGDA-Alginate
PEGDA-HA
c
Fracture Toughness (J/m²)
Hydrogel
PAAm-HA
PAAm-Chitosan
PEGDA-Alginate
PEGDA-HA

Supplementary Figure 3 | Interfacial toughness and fracture toughness of various as-prepared tough hydrogels. (**a**) Typical curves of peeling force per hydrogel width vs. displacement for various tough hydrogels bonded on PDMS substrates. (**b**) The measured interfacial toughness for various as-prepared tough hydrogels bonded on PDMS substrates based on 90-angle peeling test. (**c**) The measured fracture toughness for various as-prepared tough hydrogels based on the pure-shear test[23,43]. Values in **b**,**c** represent mean and the error bars represent standard deviation of measured interfacial toughness or fracture toughness for each tough hydrogel (n = 3-5).

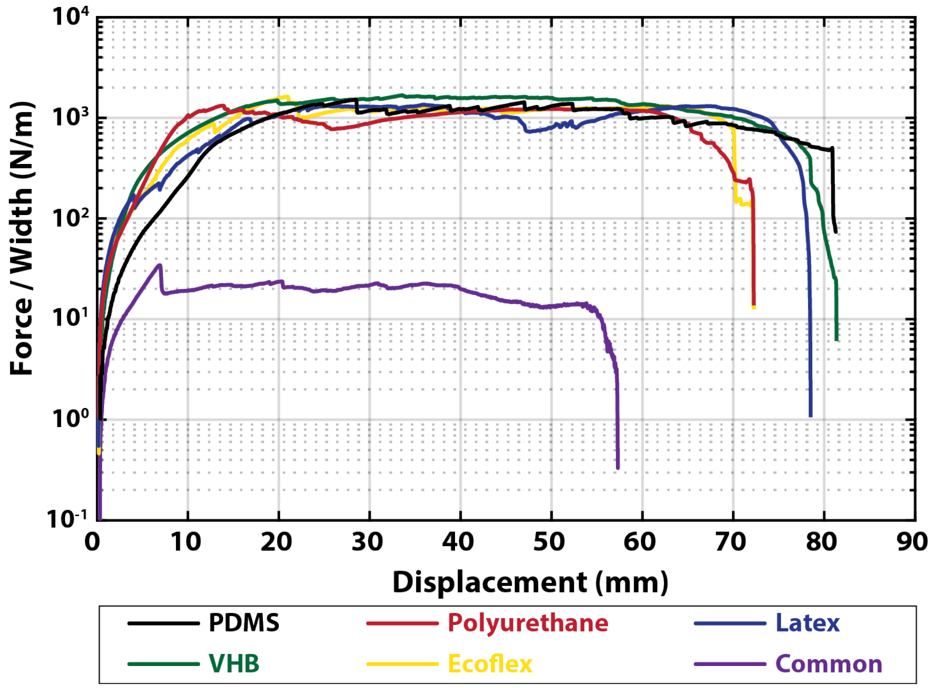

Supplementary Figure 4 | Typical curves of 90-degree peeling test for fully-swollen hydrogel bonded on various elastomers. The curves of peeling force per width of hydrogel sheet vs. displacement for various types of hydrogel-elastomer hybrids at fully-swollen state. Note that curves labeled as PDMS, polyurethane, latex, VHB™ and Ecoflex® are based on fully-swollen PAAm-alginate hydrogel bonded on these elastomers; while the curve labeled as Common is based on fully-swollen PAAm hydrogel bonded on PDMS.

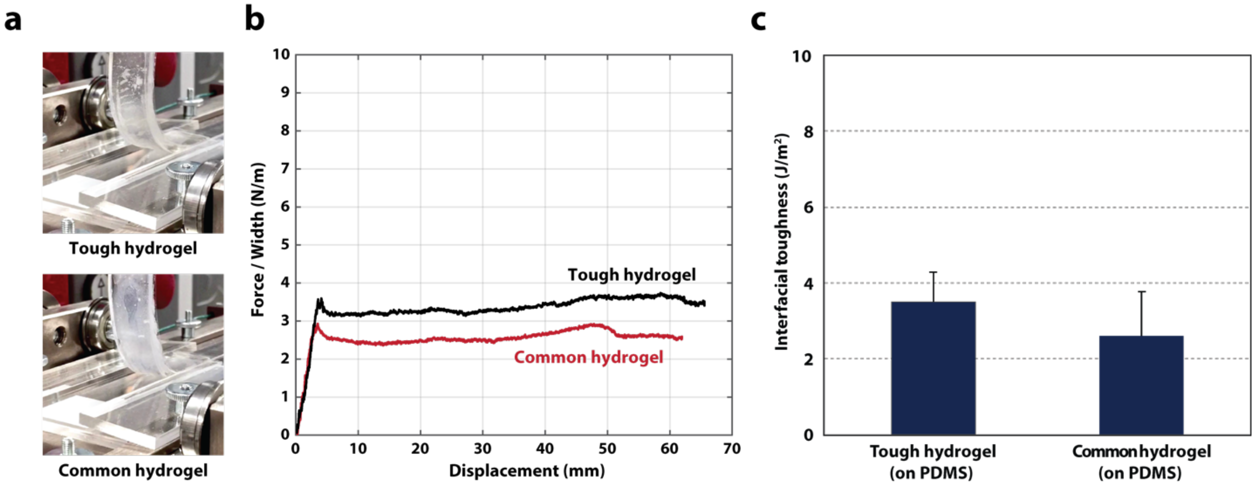

Supplementary Figure 5 | The 90-degree peeling test of hydrogels bonded on untreated elastomer substrates. (**a**) Photos of the peeling process of as-prepared PAAm-alginate tough hydrogel and PAAm common hydrogel physically-attached on PDMS substrates without benzophenone treatment on PDMS surfaces. The failure occurs at the hydrogel-elastomer interfaces. (**b**) Typical curves of peeling force per hydrogel width vs. displacement for as-prepared PAAm-alginate tough hydrogel and PAAm common hydrogel physically-attached on PDMS substrates. (**c**) The measured interfacial toughness for as-prepared PAAm-alginate tough hydrogel and PAAm common hydrogel physically-attached on PDMS substrates. Values in **c** represent mean and the error bars represent standard deviation of measured interfacial toughness for each tough hydrogel (n = 3-5).

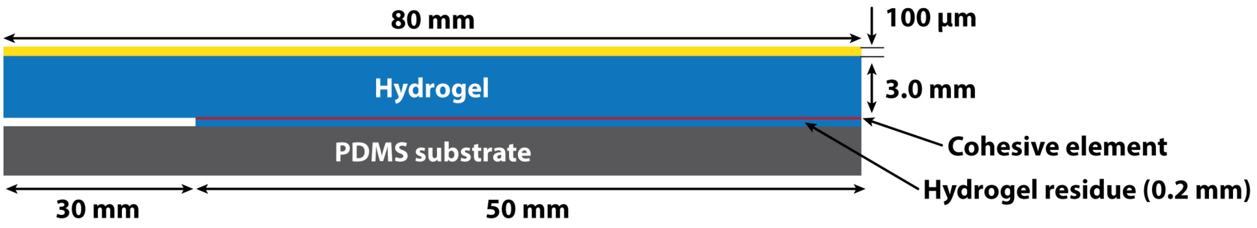

Supplementary Figure 6 | Schematic illustration of the finite-element model for numerical simulation of peeling. The yellow line indicates the stiff backing and the red line indicates the cohesive element between bulk hydrogel sheet and the thin residual hydrogel layer on various elastomer substrates.

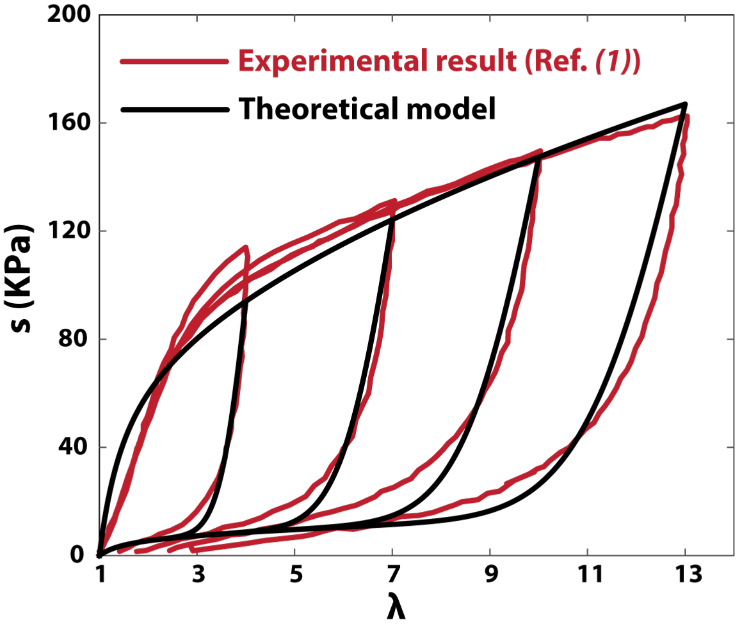

Supplementary Figure 7 | Experimental data and fitting with the Mullins effect model for PAAm-alginate hydrogel[1].

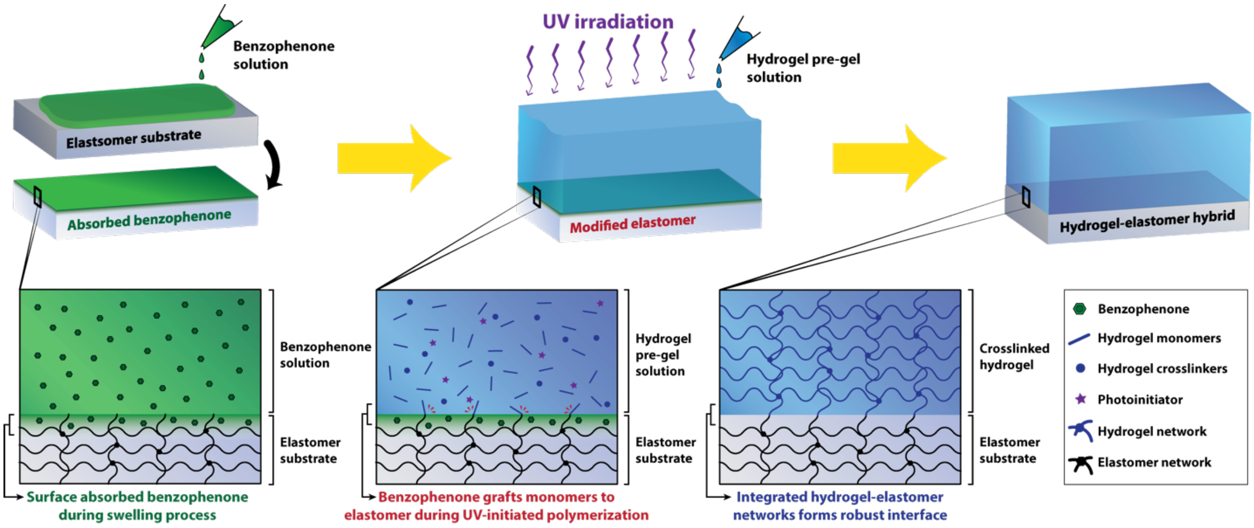

Supplementary Figure 8 | Schematics of an alternative fabrication approach for robust hydrogel-elastomer hybrid. Hydrogel can form robust bonding onto elastomers by directly curing hydrogel precursor onto benzophenone absorbed elastomer surfaces followed by UV irradiation.

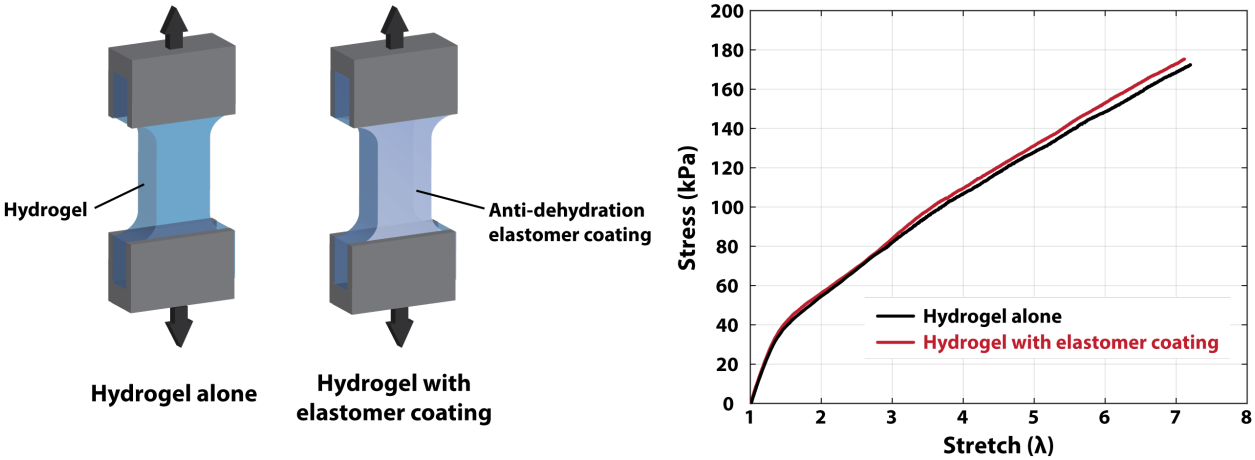

Supplementary Figure 9 | The effect of anti-dehydration elastomeric coatings on mechanical property of bulk hydrogels. Uniaxial tension is used to test PAAm-alginate hydrogels with and without the Ecoflex® anti-dehydration coatings. The stress-stretch curves of the two samples under large deformation show no significant difference, owing to the much lower thickness (100 µm) than the hydrogel, low modulus (~ 30kPa) and high stretchability (~ 7 times) of Ecoflex®.

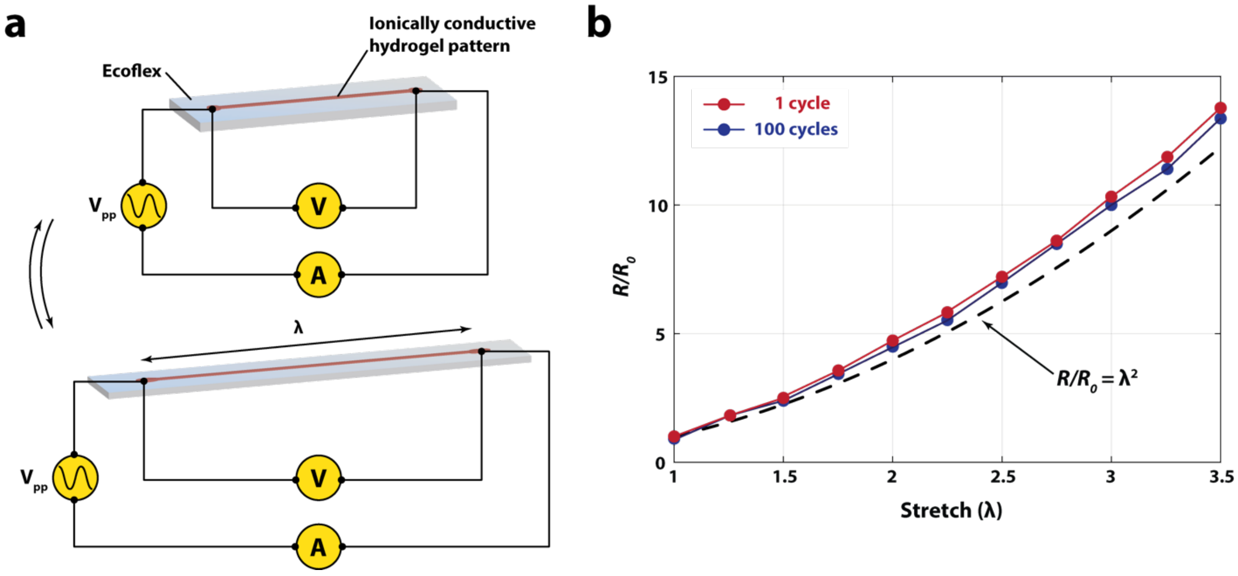

Supplementary Figure 10 | The electrical property of conductive hydrogel on elastomer under deformation. (**a**) The electrical property of ionically conductive PAAm-alginate tough hydrogels adhered on Ecoflex® is measured using four-point method. (**b**) The electrical resistance of the conductive hydrogel on Ecoflex® substrate remains almost the same after 100 cycles of stretch to 3.5 times. In addition, the relation between resistance and stretch follows $R/R_0 = \lambda^2$, where R_0 is the resistance before deformation and R is the resistance after stretch of λ from the initial state.

Supplementary Reference

1 Sun, J.-Y. *et al.* Highly stretchable and tough hydrogels. *Nature* **489**, 133-136 (2012).
